# Effects of Designer Hyper-Interleukin 11 (H11) on Hematopoiesis in Myelosuppressed Mice

**DOI:** 10.1371/journal.pone.0154520

**Published:** 2016-05-04

**Authors:** Hanna Dams-Kozlowska, Eliza Kwiatkowska-Borowczyk, Katarzyna Gryska, Anna Lewandowska, Andrzej Marszalek, Sebastian Adamczyk, Anna Kowalik, Ewa Leporowska, Andrzej Mackiewicz

**Affiliations:** 1 Chair of Medical Biotechnology, Poznan University of Medical Sciences, Poznan, Poland; 2 Department of Diagnostics and Cancer Immunology, Greater Poland Cancer Centre, Poznan, Poland; 3 Department of Oncologic Pathology and Prophylactics, Poznan University of Medical Sciences, Poznan, Poland; 4 Department of Oncologic Pathology, Greater Poland Cancer Centre, Poznan, Poland; 5 Department of Medical Physics, Greater Poland Cancer Centre, Poznan, Poland; 6 Department of Laboratory Diagnostics, Greater Poland Cancer Centre, Poznan, Poland; Emory University, UNITED STATES

## Abstract

The incidence of cancer is constantly increasing. Chemo/radiotherapy is one of major methods of treating cancer. Although adverse chemo/radiotherapy events, such as anemia and neutropenia, can be successfully cured, thrombocytopenia is still problematic. We constructed the Hyper-IL11 (H11) cytokine by linking soluble interleukin 11 receptor alpha (sIL-11Ralpha) with IL-11. *In vivo* H11 activity was examined in myelosuppressed mice. Myelosuppression was induced by either i) sublethal irradiation and carboplatin administration or ii) sublethal irradiation. A dose of 100 μg/kg of H11 or IL-11 was administered subcutaneously for 7 days. IL-11 and H11 accelerated leukocyte, hematocrit and platelet recovery. The effect on the attenuation of thrombocytopenia was significant. Moreover, both cytokines increased the cellularity and numbers of megakaryocyte, erythroid, and granulocyte/macrophage progenitors in the bone morrow and spleen compared with the control. Although H11 was administered at a molar concentration that was three times lower, its effects were comparable with or better than those of IL-11; thus, the activity of H11 was superior to that of IL-11. Because no toxicity was observed after the intravenous administration of H11, this hyper-cytokine may be potentially useful for treatment of thrombocytopenia and other IL-11-dependent disorders.

## Introduction

In 2012, noncommunicable diseases were responsible for 68% of all deaths; among them were cardiovascular diseases, cancer, diabetes and chronic lung diseases (http://www.who.int/mediacentre/factsheets/fs310/en/index2.html). Despite recent developments in cancer prevention, detection, treatment, and management, the International Agency for Research on Cancer (IARC) estimated that in 2012 there were 14.1 million new cancer cases and 8.2 million cancer deaths worldwide (http://globocan.iarc.fr/Pages/fact_sheets_cancer.aspx). By 2025 the global burden for new cancer cases, excluding non-melanoma skin cancer, is expected to grow to 19.3 million, and by 2035 to 24 million (http://globocan.iarc.fr, *GLOBOCAN 2012 (IARC)*, 28.10.2015).

The various therapies used to treat cancer include surgery, immunotherapy, chemotherapy, radiation therapy, hormonal therapy, and targeted therapy. However, they may be associated with serious adverse events, including death. A common problem of cancer chemotherapy and/or radiation therapy is the induction of myelosuppression, which results in anemia, neutropenia and thrombocytopenia. The discovery of erythropoietin and granulocyte colony-stimulating factor (G-CFS) resolved the problems of anemia and neutropenia; however, the treatment of thrombocytopenia is still an open question. Moreover, thrombocytopenia in cancer patients may be due to other factors, including infections, coagulopathy, immune thrombocytopenia, post-transfusion purpura, drugs or thrombotic microangiopathy [1, 2]. The mechanism of thrombocytopenia (reduced production, rapid demolishing or sequestration) should be taken into account when determining appropriate management of the patient.

The physiological number of platelets is 150,000–450,000/μL. Thrombocytopenia is defined as a platelet count below 150,000/μL. The National Cancer Institute (NCI) has developed the Common Toxicity Criteria to classify the severity of thrombocytopenia. Grade 1 is defined as a platelet count of <150,000 to 75,000/μL; grade 2, <75,000 to 50,000/μL; grade 3, <50,000 to 25,000/μL; and grade 4, below 25,000/μL (CTCAE Version 4.0, http://evs.nci.nih.gov/ftp1/CTCAE/About.html). An increased bleeding tendency during surgery is associated with a platelet count below 50,000/μl, and severe spontaneous life-threatening bleeding can occur with a platelet count <10,000/μl [1].

In cancer patients with a platelet count <100,000/μl, chemotherapy and radiation therapy should be administered with caution so as not to worsen the thrombocytopenia [1]. Treatment in such cases is often modified by limiting the therapeutic dose intensity or by delaying a dose. Unfortunately, this can ultimately compromise the cancer treatment outcome. Platelet transfusion, the standard treatment for thrombocytopenia, is associated with risks such as alloimmunization, transmission of infection, transfusion reactions, and platelet refractoriness [3]. Moreover, the procedure is costly, and platelet resources are limited. In some cases, repeated transfusions are needed. Additional healthcare costs may be associated with frequent laboratory examinations and additional clinic visits [4]. Despite these problems, the number of platelet transfusions has gradually increased. Because the number of new cancer cases is expected to grow, there is high demand for therapies to treat cancer-related thrombocytopenia.

Because thrombopoietin (TPO) is a major factor during megakaryopoiesis, it is considered a potent agent for the treatment of thrombocytopenia. The first generation of thrombopoietic agents was recombinant versions of TPO; rhTPO was a full-length molecule, and PEG-rHuMGDF was a truncated, pegylated version of TPO [3, 5, 6]. Unfortunately, the clinical development of these agents was halted after neutralizing antibodies were found in healthy subjects; they caused thrombocytopenia in healthy volunteers [7, 8]. Recently, a next generation of TPO mimetic factors was developed [3, 5, 9]. Two of them, romiplostim and eltrombopag, were approved by the Food and Drug Administration (FDA) for the treatment of immune thrombocytopenia and hepatitis C-related thrombocytopenia [9, 10]. Several preliminary reports of small phase I/II studies have indicated their safety and partial efficacy in chemotherapy-related thrombocytopenia [11, 12].

In addition to TPO, other agents, such as interleukin 1 (IL-1), IL-6, IL-3 and IL-11, have been studied in clinical trials as thrombocytopenia agents [13]. Although most of them have shown promising thrombopoietic activity, their use has been limited because of their toxicity. IL-11 is the only agent approved by the FDA in the United States to prevent severe thrombocytopenia and reduce the need for platelet transfusion following myelosuppressive chemotherapy for non-myeloid malignancies [14]. IL-11 proved its effectiveness in treatment of thrombocytopenia; however, it causes adverse events, which limits its use [10, 15].

Hyper**-**IL-11 (H11) is a designer cytokine composed of full-length soluble IL-11 receptor (sIL-11R) and IL-11 [16]. It comprises two naturally existing components bound without artificial linker. The bioactivity of H11 has been demonstrated in various experimental models *in vitro* [16, 17] and *in vivo* [18, 19], showing that a lower effective dose of H11 was needed to support its bioactivity than with IL-11 alone.

Previously, we showed that H11 stimulated hematopoiesis and was more effective than IL-11 in enhancing proliferation of early progenitors and directing them to megakaryocyte (Mk) and erythroid cells and in inducing Mk maturation *in vitro* [17]. In the present study, we evaluated *in vivo* H11 activity in hematopoiesis. Mice were exposed to chemotherapy and/or radiation for the induction of myelosuppresion and then treated with IL-11, H11 and PBS as a vehicle control. The systemic and cellular effects were examined. The obtained results demonstrated that H11 shows therapeutic activity in a mouse model of myelosuppression and that its activity is higher than that of IL-11.

## Materials and Methods

### Animals

Six-week-old female BALB/cAnNCrl mice were purchased from Charles River Laboratories International, Inc. (Erkrath, Germany). The animals were kept under constant pathogen-free conditions with a 12-hour day/night cycle and unlimited access to food and water. Mice were used at the age of 9–10 weeks. All experiments were performed according to the national and institutional guidelines for the humane treatment of laboratory animals after approval by the Local Ethical Committee for the Experiments on Animal in Poznan (Permit Number 62/2011). *A*ll efforts were made to minimize animals suffering.

### Recombinant cytokines

Recombinant H11 was produced in a Baculovirus expression system and then was purified as described previously [16]. The recombinant cytokines rhIL-11, rhTPO, rhIL-6, rmIL-3 were purchased from ImmunoTools, Friesoythe, Germany.

### Myelosuppressive regimen and treatment

Two types of myelosuppressive regimens were used: i) 500 cGy whole-body irradiation in combination with carboplatin administration and ii) 300 cGy whole-body irradiation. Clinac 2300 C-D\S linear accelerator (Varian Medical Systems, Palo Alto, CA) was used as a source of 500 cGy irradiation at a dose rate of <100 cGy/min. In this regimen, irradiation was followed by a single intraperitoneal injection of 1.2 mg/mouse of carboplatin (medac GmbH, Wedel, Germany). RS 2000 irradiator (Rad Source Technologies, Inc., Suwannee, GA) was used for 300 cGy irradiation at a dose rate of 103.6 cGy/min. After myelosuppression induction, mice were divided into 3 groups (each of 15 animals): treated with IL-11, treated with H11 or served as the vehicle control (treated with PBS). Mice subcutaneously received 100 μg/kg/day of each cytokine in 100 μl sterile PBS (Sigma-Aldrich Co., St. Louis, MO). The injections were performed for seven consecutive days, starting the day after irradiation (the first day of the experiment).

### Hematology

Peripheral blood was collected by a retro-orbital puncture into heparinized tubes on days 3, 7, 10, 15, 20 and 30 (or as indicated) following irradiation. Five mice from each experimental group were bled on designated days such that no individual mouse was bled more than once per week. White blood cells (WBC), red blood cells (RBC), hemoglobin, hematocrit (HCT) and platelets (PLT) were automatically counted within 20 minutes after blood collection using an ABC Vet Automated Blood Counter (Scil Animal Care Company GmbH, Viernheim, Germany) with mouse-specific discriminator settings.

### Preparation of bone marrow and spleen cells for colony-forming cell assays

Mice (5 animals per group per time-point) were sacrificed on days 10, 15 and 30 of the experiment (or as indicated); two femurs and the spleen were collected from each mouse. Whole bone marrow was harvested from the femurs by flushing the marrow cavity with X-Vivo 10 medium (Lonza, Verviers, Belgium). To isolate splenocytes, spleens were pushed through a sterile 70 μm cell strainer (Corning Incorporated, Corning, NY) in PBS. Single-cell suspensions of bone marrow or splenocytes were obtained by passing cells through an 18-gauge needle. The cells were centrifuged at 350 x g for 10 min. Erythrocytes were removed by lysis in hypotonic ammonium chloride (AKC lysing buffer) for 5 min at room temperature. The cells were washed twice in PBS, and after centrifugation, were resuspended in X-Vivo 10 medium supplemented with 1x Antibiotic Antimycotic Solution (Sigma-Aldrich Co., St. Louis, MO) passed over a 70 μm nylon filter (BD Biosciences, San Jose, CA) and counted with a Fuchs-Rosenthal counting chamber. Viability was accessed by trypan blue staining. Solutions of 1 x 10^7^ cells/ml (or as indicated) were used for colony-forming cell assays.

### Colony-forming cell (CFC) assay

For determination of the number of erythroid, granulocyte/macrophage and multi-potential progenitors, a Mouse CFC Assay using MethoCult GF M3434 methylcellulose medium with recombinant cytokines for mouse cells (StemCell Technologies, Vancouver, Canada) was used according to the manufacturer’s instructions. Briefly, a 10 x concentrated cell suspension (2 x 10^5^/ml and 1 x 10^6^/ml of bone marrow and spleen cells, respectively) was prepared in X-Vivo10 (Lonza, Verviers, Belgium) medium. A total of 110 μl of 10 x concentrated cells was mixed with 1.1 ml of MethoCult medium and was plated on a 35 mm culture dish. The cell cultures were maintained at 37°C in a 5% CO_2_/95% air atmosphere of 95% humidity for 12 days. Additional water dishes were provided to maintain humidity. The erythroid (erythroid burst-forming units, BFU-E), granulocyte-macrophage (granulocyte-macrophage colony-forming units, CFU-GM) and multipotential (granulocyte erythrocyte macrophage megakaryocyte colony-forming units, CFU-GEMM) progenitor cells were analyzed using an inverted light microscope.

### Quantitation of megakaryocytic progenitors

Bone marrow and spleen megakaryocyte progenitors were quantitated using a MegaCult-C system (StemCell Technologies, Vancouver, Canada) according to the manufacturer’s instructions. Briefly, a 33 x concentrated cell suspension (4.4 x 10^6^/ml and 1 x 10^7^/ml of bone marrow and spleen cells, respectively) was prepared in X-Vivo10 medium. To the 1 ml of MegaCult-C medium containing cytokines (rhTPO (50 ng/ml), rhIL-6 (20 ng/ml), rhIL-11 (50 ng/ml) and rmIL-3 (10 ng/ml)), 50 μl of 33 x concentrated cell suspension and 0.6 ml of cold collagen solution (3 mg/ml) were added. From the final culture mixture, 0.75 ml was dispensed into each of two wells of a double-chamber slide. Slides were placed inside a covered 100 mm Petri dish with an additional water container to maintain humidity; the slides were incubated for 8 days at 37°C in a 5% CO_2_/95% air atmosphere of 95% humidity. The megakaryocytes were stained for acetylcholinesterase according to the manufacturer’s instructions (StemCell Technologies, Vancouver, Canada). The cells were counterstained with Harris’ hematoxylin solution for 30 seconds. The megakaryocyte (megakaryocyte colony-forming units, CFU-Mk) progenitor, which was identified as a cluster of cells containing three or more positively stained cells, was scored under a light microscope.

## Histology

**Fig 1:**
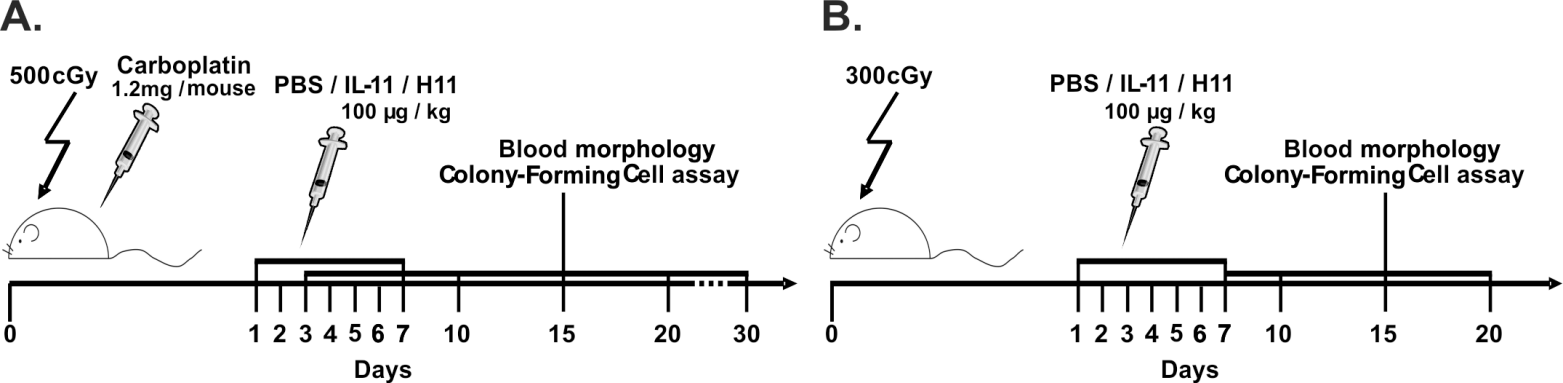
**Applied myelosuppressive regimen models: (A) chemo/radiotherapy model—whole-body irradiation in combination with carboplatin administration and (B) radiotherapy model—whole-body irradiation.** After myelosuppression, the mice were treated with PBS (vehicle control), IL-11, or H11 for seven consecutive days. On days (**A**) 3, 7, 10, 15, 20 and 30 or (**B**) 7, 10, 15 and 20, the peripheral blood was hematologically analyzed. On days (**A**) 10, 15 and 30 or (**B**) 10 and 15, the bone marrow and spleen cells were isolated for progenitor quantification.

The mice were killed 10 days after irradiation and treatment according to the scheme shown in Fig 1B. Organs such as the bone marrow (sternum), spleen, lung and liver were fixed in 10% buffered formalin. Next, the samples were processed routinely for paraffin embedding, and after sectioning at a thickness of 4.5 μm, the samples were stained with hematoxylin and eosin (HE) for light microscope evaluation.

The evaluation of bone marrow cellularity was based on an analysis of a relation of haematopoietic cells to adipose tissue within the bone marrow. It is one of the basic elements of diagnostics procedure in haematology and it is examined on the base of the expertise of observer. The bone marrow cellularity were an estimated values assessed during analysis of all samples available within a given group.

To determine the number of Mks in the bone marrow, the three fields of highest density of Mks (hot spots) were selected on each specimen and the number of Mks was counted. The samples from three mice for each group were examined.

The extent of fibrosis in the spleen was quantified by measuring the surface area of the fibrous tissue in four different locations of its highest intensity (hot spots). The surface area of tissue was 0.556 mm^2^ (the micrographs of hot spots were taken at the objective 10X). The samples from three mice for each group were examined. The surface area was quantified using program CellSens (Olympus Coropration, Center Valley,PA). The obtained values (in square millimetres) were used to calculate the means and standard deviations of surface area of one single spot for all groups and means and standard deviations of total surface area of all measured fibrosis spots for each group.

### Biochemical analysis of serum

Ten days after irradiation and treatment according to the scheme shown in Fig 1B, blood for the serum evaluation was collected by retro-orbital puncture. Alanine transaminase (ALT), aspartate transaminase (AST) and lactate dehydrogenase (LDH) were measured using ALTL, ASTL, LDHI2 assays, respectively (Roche Diagnostics GmbH, Mannheim, Germany), and a Cobas 6000 biochemical analyzer (Roche Diagnostics GmbH, Mannheim, Germany).

### Statistics

To test for significant differences between the PBS-, IL-11- and H11-treated groups, one-way ANOVA was used. In cases of significance, ANOVA (p<0.05) post hoc tests with a Bonferroni correction were performed. Differences were considered significant when p<0.05.

## Results

### Two myelosuppressive regimen models

To study the hematopoietic activity of H11 *in vivo*, two myelosuppressive regimen models were applied: i) a model induced by sublethal irradiation and chemotherapeutic administration and ii) a model induced by a lower dose of irradiation (Fig 1). Because applied models may reflect treatment during cancer therapy, they were named chemo/radiotherapy and radiotherapy induced myelosuppression, respectively. The radiotherapy model was used for better comparisons of H11 to the control groups because the observed mortality in the PBS group in the chemo/radiotherapy model made statistical analysis impossible. For 7 days, mice received the same dose of IL-11 or H11; the H11 dose that was administered per animal was three times lower at the molar level than the amount of IL-11. Age-matched naïve mice (control) were used for comparisons with the myelosuppressed mice.

### Hematology after H11 treatment

**Fig 2:**
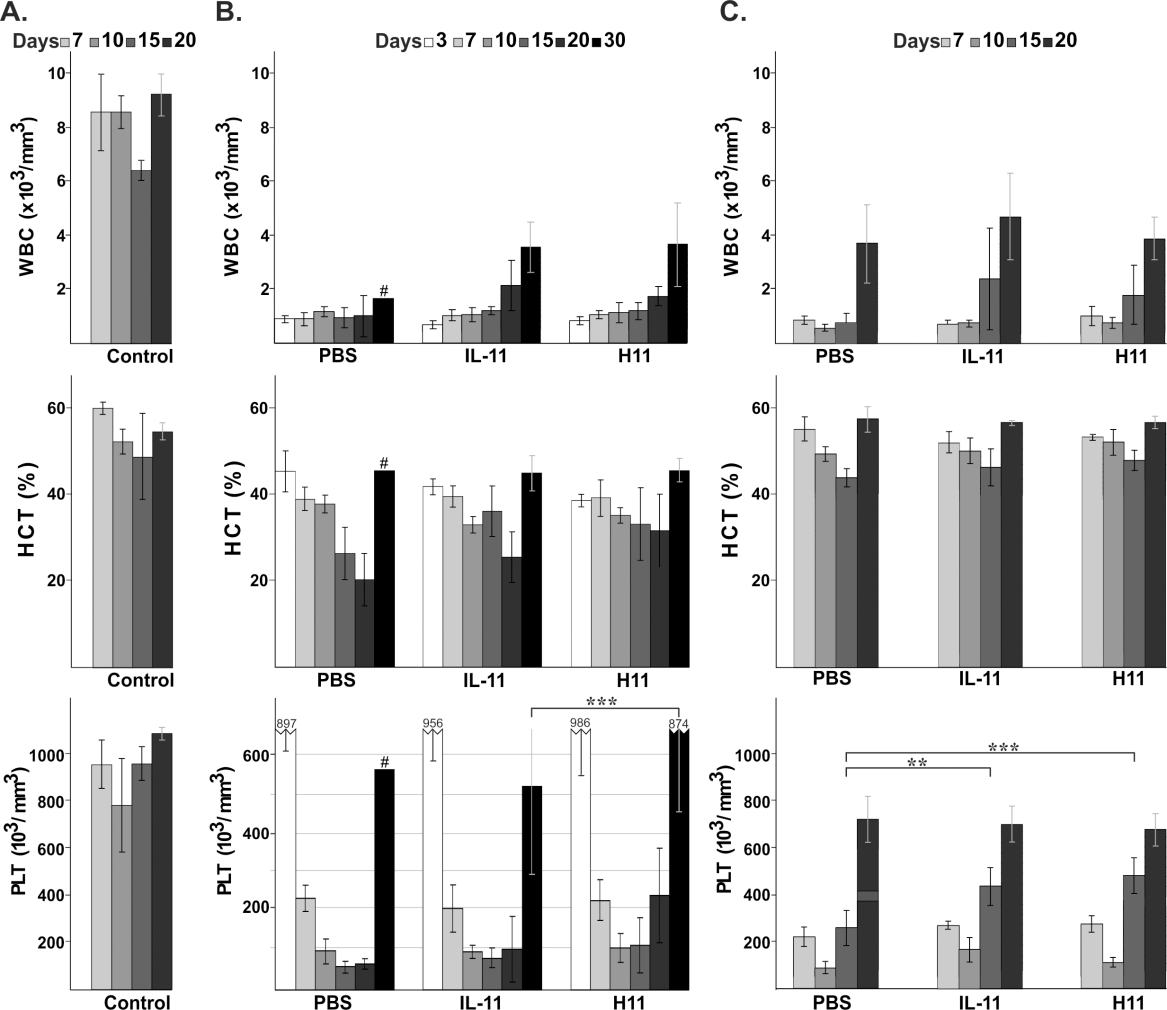
**Effect of H11 on the number of white blood cells (WBC), hematocrit (HCT) and platelets (PLT) after (B) chemo/radiotherapy or (C) radiotherapy induced myelosuppression compared with vehicle (PBS) and IL-11-treated mice and (A) age-matched naïve mice (control).** The data represent the mean +/- SD from 5 mice per each time point except for the results indicated by a # sign, which represent the average data from two animals. *** indicates statistical significance with p<0.001 and ** p<0.01.

The application of sublethal irradiation and a chemical agent (Carboplatin) resulted in severe leukopenia, thrombocytopenia and decreased hematocrit (Fig 2B). The PBS-treated group suffered from severe leukopenia for 30 days of monitoring, and on the last day of the experiment, only two animals had survived. On day 15, the WBC count was 30% higher in the groups receiving IL-11 and H11 than in the PBS group, and on day 30, the leukocyte count was two-fold higher in the mice treated with both cytokines than in the surviving animals that were treated with the vehicle. The nadir of the hematocrit occurred on day 20 in all treated groups; however, in the IL-11 and H11 administered mice, it was less severe at 30% and 60% above the vehicle-treated group, respectively. On day 30, there was no difference in the hematocrit between the groups. The RBC counts in all groups of mice were consistent with the changes observed in hematocrit (data not shown). After chemo/radiotherapy treatment, the mice developed severe and prolonged thrombocytopenia. The PLT count nadir occurred in the PBS- and IL-11-treated groups on day 15, but in the H11 group, it occurred earlier (on day 10). Fifteen days after the induction of myelosuppression, the platelet counts in the IL-11- and H11-treated mice were approximately 45% and 100% above the PBS group, respectively. On day 20, they were approximately 1.60- and 3.7-fold higher in the IL-11 and H11 groups, respectively, compared with the vehicle-treated mice. On the last day of the experiment (day 30), the platelet counts in the H11 group were 1.65 times higher than in the IL-11-treated group. The difference was significant. The mean PLT count of the best responders (two survived animals) in the PBS group was comparable with that of the IL-11-treated mice.

Single irradiation resulted in less severe myelosuppression than chemo/radiotherapy treatment (Fig 2C). The radiation caused severe leukopenia in all groups of animals. The WBC count in the PBS group increased at the last examined time point (day 20), whereas in the IL-11 and H11-treated groups, the WBC increased earlier (day 15). At that time point (day 15), the leukocyte count was approximately 2-fold higher in the IL-11 and H11 groups than in the vehicle-treated mice. A similar decrease in hematocrit was observed in all examined groups; however, its nadir occurred earlier (day 15 vs. 20, respectively), and it was less severe (45% vs. 20–30%, respectively) than in the respective chemo/radiotherapy groups (Fig 2C vs. 2B, middle panels, respectively). On day 20, it recovered to a normal level in all mice. Thrombocytopenia was pronounced in all treated groups; however, it was not prolonged. The platelet number on day 7 was similar for all groups and on day 10 the nadir of platelets occurred for all treated animals. On day 15, an approximately 2-fold increase in platelet count was observed in the IL-11 and H11-treated groups compared with the PBS mice. The differences were significant. At the last time point of the experiment (day 20), all animals exhibited recovered platelet counts.

### Effect of H11 on the number of bone marrow and spleen cells

**Fig 3:**
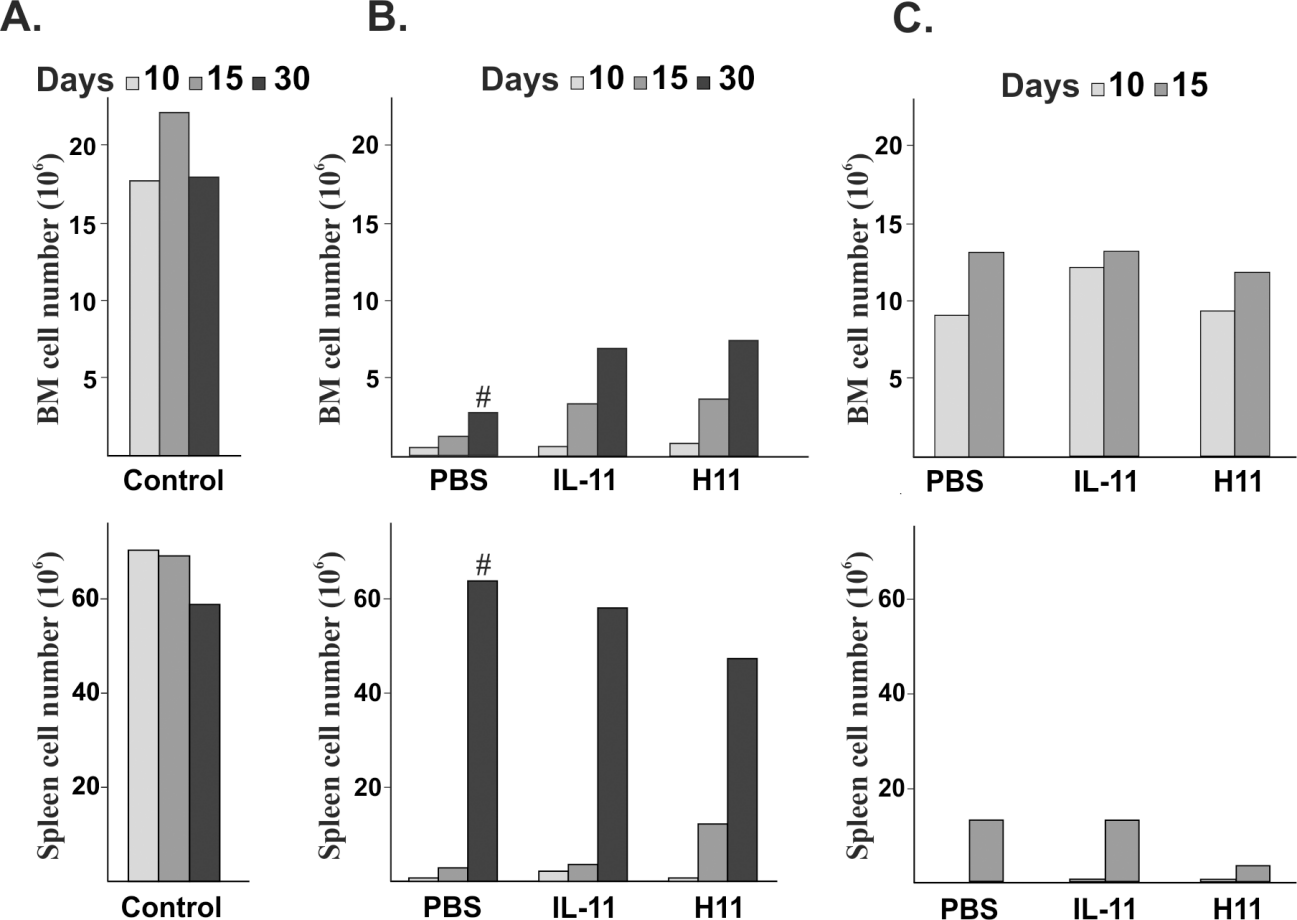
**Effect of H11 on the number of bone marrow and spleen cells after (B) chemo/radiotherapy or (C) radiotherapy induced myelosuppression compared with vehicle (PBS), IL-11-treated mice and (A) age-matched naïve mice (control).** The bone marrow and spleen cells were pooled from 5 animals per each time point, and the data are shown as the mean number of cells per animal, except for the results indicated by a # sign, which represents the average data from two animals.

The applied chemo/radiotherapy and radiotherapy models resulted in the severe reduction of live cells isolated from bone marrow (from femur) and spleen (Fig 3). On day 10, we isolated approximately 0.66 x 10^6^ bone marrow cells per femur from chemo/radiotherapy groups, whereas from the spleen, we isolated 0.1 x 10^6^ of live cells from all groups of mice. At the same time point, approximately 10x 10^6^ of bone marrow cells per femur from irradiated animals in all treated groups and individual, 0.25, and 0.33 x 10^6^ of live cells per spleen were isolated from the PBS, IL-11 and H11 groups, respectively. The application of IL-11 and H11 after chemo/radiotherapy accelerated the cellularity recovery of the bone marrow and spleen cells compared with the vehicle-treated mice (Fig 3B). For bone marrow recovery, cytokine application resulted in a more pronounced effect. The effect of IL-11 and H11 on the recovery of bone marrow and spleen cellularity after a single irradiation was negligible or even inhibitory (on day 15, the number of spleen cells derived from the H11 group was approximately 4-fold lower than that derived from the PBS mice) (Fig 3C).

### Quantitation of bone marrow progenitors after H11 administration

Hematopoietic progenitors were analyzed in bone marrow on days 10, 15 and 30 after chemo/radiotherapy treatment (Table 1). On day 10, colony-forming cells (CFCs) in the PBS group were not observed, but singular and a few progenitors were detected in the IL-11- and H11-treated mice, respectively. On day 15, IL-11 and H11 stimulation resulted in a substantial increase in CFC numbers relative to the vehicle-treated animals, and the total number of CFCs was higher in the H11 group compared with the IL-11 group (42 vs. 31, respectively). After 30 days of myelosuppression induction in the H11 treated mice, the number of hematopoietic progenitors was twofold higher than in the IL-11-treated group.

**Table 1:** The effect of H11 on the numbers of bone marrow BFU-E, CFU-GM, CFU-GEMM and CFU-Mk compared with the age-matched naïve mice (control), vehicle- (PBS) and IL-11-treated mice after chemo/radiotherapy.

Days	Treatment	Colonies				
		BFU-E^1^	CFU-GM^1^	CFU-GEMM^1^	CFU-MK^2^	Total
10	PBS	0	0	0	0	0
	IL-11	1	0	0	0	1
	H11	1	4	0	1	6
	Control	21	49	8	12	90
15	PBS	0	0	0	2	2
	IL-11	3	23	1	4	31
	H11	8	23	2	9	42
	Control	18	39	6	18	81
30	PBS	ND	ND	ND	ND	ND
	IL-11	3	33	0	8	44
	H11	6	62	3	13	84
	Control	8	50	5	19	82

^1^ Colony Forming Cell (CFC) assay: number of colonies per 2 x 10 ^4^ of bone marrow cells pooled from 5 animals

^2^ Megakaryocyte Colony Forming Cell assay: number of colonies per 1 x 10 ^5^ of bone marrow cells pooled from 5 animals

ND–Not Determined

A single irradiation caused less severe damage in bone marrow progenitors than chemo/radiotherapy treatment (Table 2). On day 10, a few CFCs were observed in the PBS group, whereas IL-11 and H11 administration resulted in 3-fold and 4-fold increases in progenitor numbers relative to the vehicle-treated mice, respectively. On day 15, the total number of CFCs in IL-11 and H11 groups was elevated compared with normal mice (control) and was approximately two-fold higher than in the PBS group.

**Table 2:** The effect of H11 on the numbers of bone marrow BFU-E, CFU-GM, CFU-GEMM and CFU-Mk compared with the age-matched naïve mice (control), vehicle- (PBS) and IL-11-treated mice after radiotherapy administration.

Days	Treatment	Colonies				
		BFU-E^1^	CFU-GM^1^	CFU-GEMM^1^	CFU-MK^2^	Total
10	PBS	2	4	0	1	7
	IL-11	3	12	3	2	20
	H11	10	18	4	1	33
	Control	16	48	5	14	83
15	PBS	6	27	1	0	34
	IL-11	20	70	1	1	92
	H11	13	60	1	16	90
	Control	9	55	3	20	87

^1^ Colony Forming Cell (CFC) assay: number of colonies per 2 x 10 ^4^ of bone marrow cells pooled from 5 animals

^2^ Megakaryocyte Colony Forming Cell assay: number of colonies per 1 x 10 ^5^ of bone marrow cells pooled from 5 animals

### Quantitation of spleen progenitors after H11 administration

Ten days after myelosuppression induced by chemo/radiotherapy, BFU-E, CFU-GM, and CFU-GEMM were not observed in the spleen, but the number of Mk progenitors was elevated in all groups (Table 3). Treatment with IL-11 and H11 increased the number of Mk colonies by 2.4- and 1.6-fold, respectively, compared with the PBS group. On day 15, a few hematopoietic progenitors were observed in cytokine-administered mice, but on day 30, the total number of CFCs was approximately 4- and 5-fold higher in the IL-11 and H11 groups, respectively, than in the control mice. The number of Mk progenitors was considerably elevated until the last day of the experiment relative to the control group, reaching the highest number of megakaryocytic colony on day 15 for IL-11 treatment, and on day 30, for the H11 mice.

**Table 3:** The effect of H11 on the numbers of spleen BFU-E, CFU-GM, CFU-GEMM and CFU-Mk compared with the age-matched naïve mice (control), vehicle- (PBS) and IL-11-treated mice after chemo/radiotherapy.

Days	Treatment	Colonies				
		BFU-E^1^	CFU-GM^1^	CFU-GEMM^1^	CFU-MK^2^	Total
10	PBS	0	0	0	19	19
	IL-11	0	0	0	45	45
	H11	0	0	0	30	30
	Control	7	21	1	8	37
15	PBS	0	1	0	33	34
	IL-11	0	5	1	80	86
	H11	0	2	1	29	32
	Control	11	12	1	6	30
30	PBS	ND	ND	ND	ND	ND
	IL-11	8	73	2	30	113
	H11	13	81	6	57	157
	Control	5	13	1	6	25

^1^ Colony Forming Cell (CFC) assay: number of colonies per 1 x 10 ^5^ of spleen cells pooled from 5 animals

^2^ Megakaryocyte Colony Forming Cell assay: number of colonies per 2.25 x 10 ^5^ of spleen cells pooled from 5 animals

ND–Not Determined

Radiotherapy caused severe damage to the spleen because BFU-E, CFU-GM, CFU-GEMM were not observed in the IL-11 and H11 groups. Moreover, analysis on day 10 was not possible in PBS mice because of a very small number of living splenocytes (Table 4). However, the number of Mk progenitors was elevated compared with the control (naïve) group (4- and 5-fold higher in the IL-11 and H11 groups, respectively). On day 15, the total number of CFCs increased in all groups relative to the results obtained on day 10, and for the IL-11-treated mice, this increase was approximately 2-fold higher than for other groups. Treatment with IL-11 and H11 resulted in a substantially higher number of CFU-Mk compared with control and PBS groups.

**Table 4:** The effect of H11 on the numbers of spleen BFU-E, CFU-GM, CFU-GEMM and CFU-Mk compared with the age-matched naïve mice (control), vehicle- (PBS) and IL-11-treated mice after radiotherapy administration.

Days	Treatment	Colonies				
		BFU-E^1^	CFU-GM^1^	CFU-GEMM^1^	CFU-MK^2^	Total
10	PBS	ND	ND	ND	ND	ND
	IL-11	0	0	0	21	21
	H11	0	0	0	25	25
	Control	10	20	2	5	37
15	PBS	5	12	4	2	23
	IL-11	23	37	2	30	92
	H11	10	17	1	22	50
	Control	11	13	1	3	28

^1^ Colony Forming Cell (CFC) assay: number of colonies per 1 x 10 ^5^ of spleen cells pooled from 5 animals

^2^ Megakaryocyte Colony Forming Cell assay: number of colonies per 2.25 x 10 ^5^ of spleen cells pooled from 5 animals

ND–Not Determined

### Histopathological analysis of organs after H11 administration

**Fig 4:**
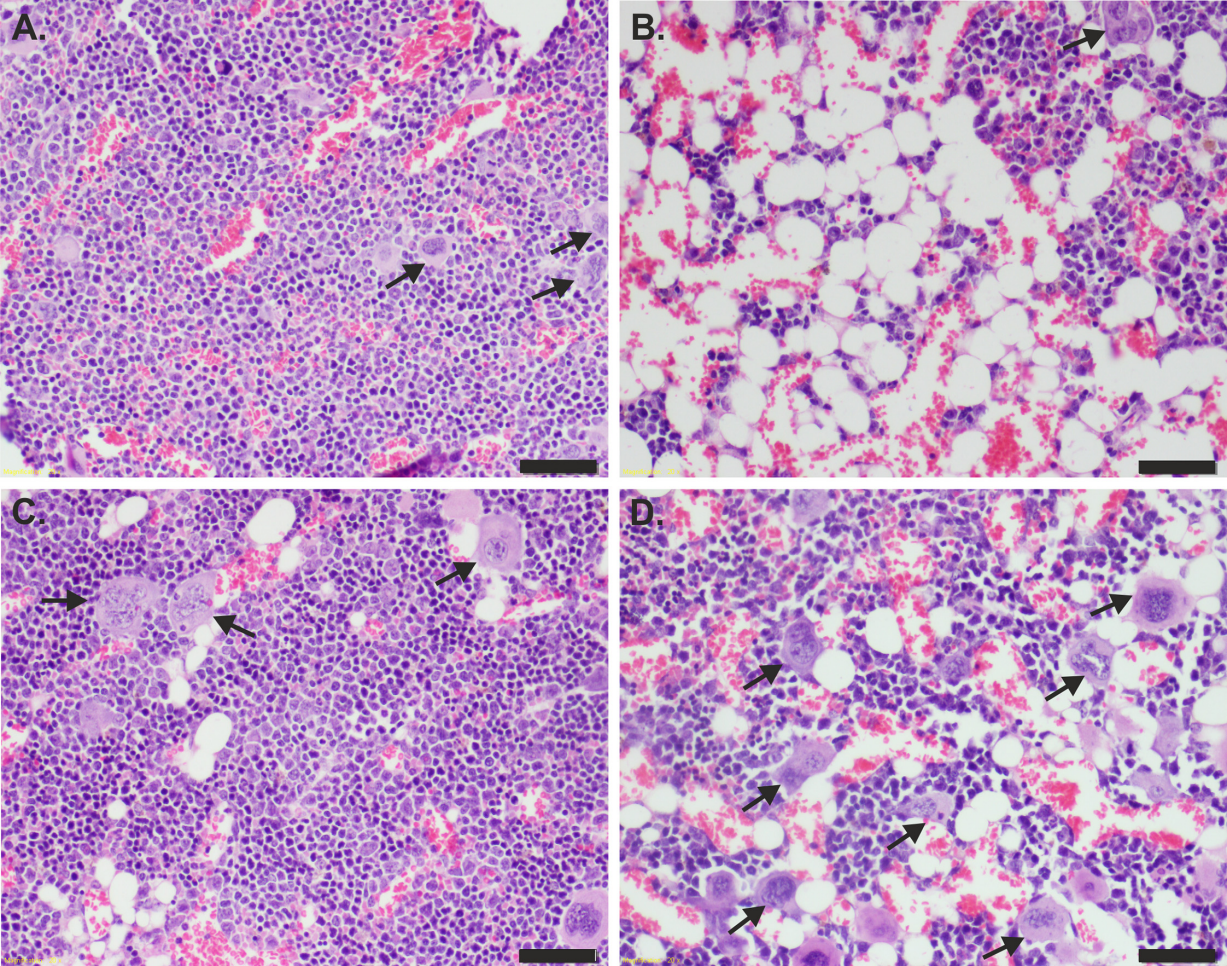
**Histology of bone marrow (sternum) of (A) age-matched naïve mice (control) and mice irradiated and then treated with (B) PBS, (C) IL-11 and (D) H11.** Ten days after treatment, the bone marrow specimens were HE stained and examined under light microscopy (magnification 20X). Arrows denote megakaryocytes. Scale bar represents 50 μm.

Ten days after irradiation, the bone marrow (sternum), spleen, liver and lungs were examined under light microscope. The cellularity of the bone marrow was decreased in all irradiated mice compared with the naïve (control) animals (Fig 4). However, there was a difference in the percentage of hematopoietic cells between the groups. The control group demonstrated 90% hematopoietic cells occupying the space within the bone marrow, whereas in the IL-11, H11 and PBS groups, 70%, 60–70% and 40–50%, respectively, hematopoietic cells were observed. In the animals receiving IL-11 and H11, an increased percentage of immature forms of the granulocyte line relative to the PBS and control groups was observed. Moreover, the ratio of the erythrocytic:granulocytic lines was altered. Namely, the ratio was 1:4, 1:5 and 1:7 in the PBS, IL-11 and H11 groups, respectively, compared with the control group (1:3). Moreover, the mean number of Mk cells were examined for all groups indicating the highest number of Mks for H11 treated mice (Table 5). The difference of mean number of Mks between PBS and H11 groups was significant.

**Table 5:** The effect of H11 on the number of bone marrow Mk cells compared with the age-matched naïve mice (control), vehicle- (PBS) and IL-11-treated mice after a single irradiation.

Treatment	Mean Number of Mk cells (SD)
PBS	3.8 (2.2)
IL-11	6 (3.4)
H11	11.7 (2.6)*
Control	5.3 (2.3)

The number of Mks in the bone marrow was determined by by histopathological examination of the samples. The means and SD from three mice per group (three spots for each mouse) are shown.

* indicates statistical significance with p<0.05.

**Fig 5:**
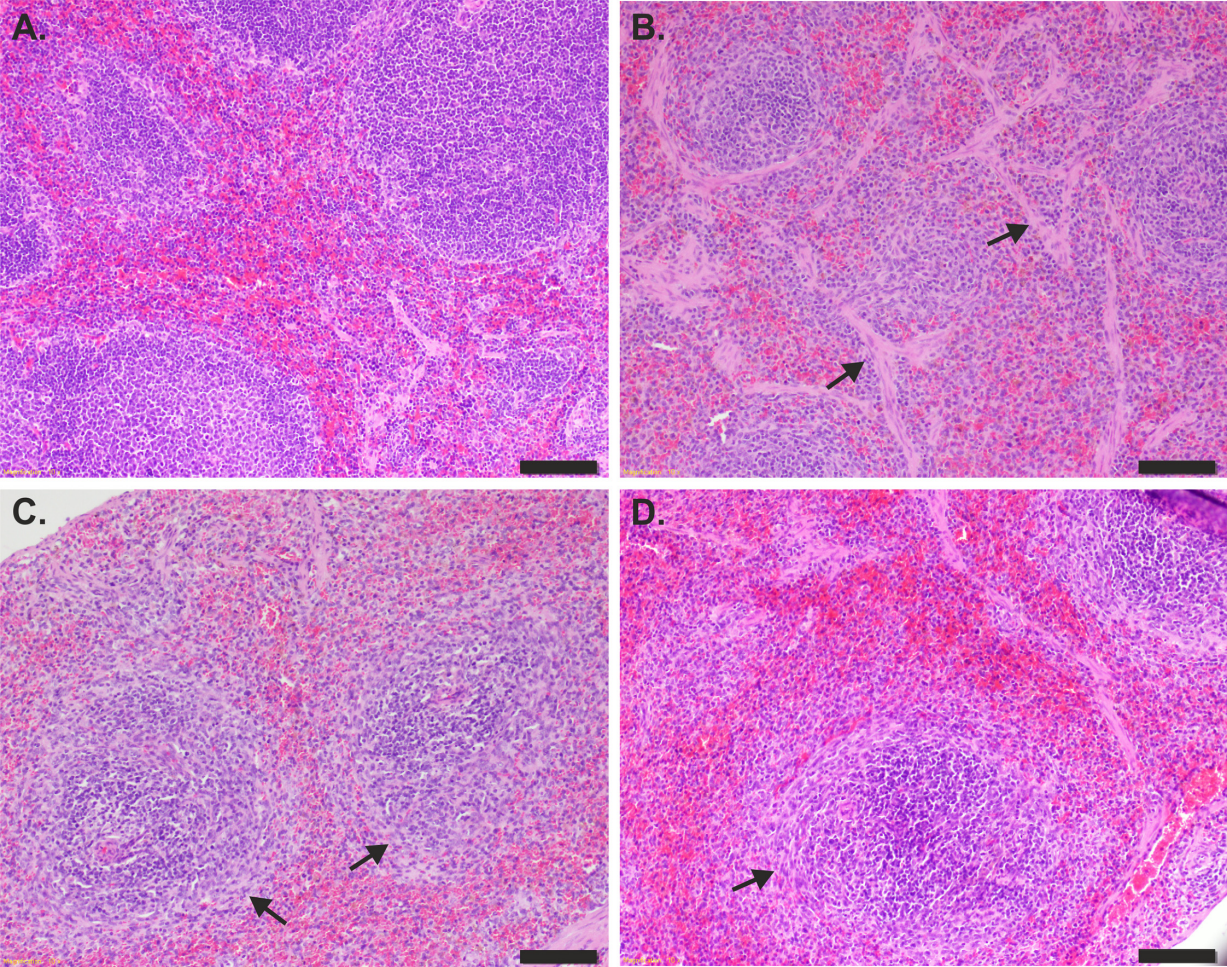
**Histology of the spleen of (A) the age-matched naïve mice (control) and the mice irradiated and then treated with (B) PBS, (C) IL-11 and (D) H11.** Ten days after treatment, spleen specimens were HE stained and examined under light microscopy (magnification 10X). Arrows denote fibrosis. Scale bar represents 100 μm.

The spleen parenchyma revealed fibrosis in all irradiated animal groups (Fig 5); however, the intensity of the fibrosis differed. The extent of fibrosis was quantified by measuring the surface area of the fibrous tissue in four different locations for each sample for three mouse in each group (S1 Fig). The mean of surface area of one single spot of fibrosis was the highest for PBS group comparing with IL-11 and H11 treated groups and control mice (Table 6). The mean of total surface area of all measured fibrosis spots for PBS treated group was three times higher than in all other groups and the difference was significant (PBS vs IL-11 and H11 groups). There was no significant difference between effect of IL-11 and H11 treatment. Moreover, features of hyperemia and hemosiderin deposits were observed in the spleen from all irradiated mice groups (Fig 5).

**Table 6:** The effect of H11 on the intensity of the fibrosis of spleen compared with the age-matched naïve mice (control), vehicle- (PBS) and IL-11-treated mice after a single irradiation.

Treatment	Surface area of fibrosis (mm^2^)	
	Mean of single fibrosis spot (SD)	Mean of total area of measured fibrosis spots (SD)
PBS	0.013 (0.007)	0.220 (0.064)
IL-11	0.008 (0.006)	0.073 (0.047)*
H11	0.006 (0.003)	0.072 (0.039)*
Control	0.007 (0.004)	0.073 (0.038)

The intensity of the fibrosis of spleen was determined by measurement the surface area of fibrosis in HE specimens. The means and SD from three mice per group (four spots for each mouse) are shown.

* indicates statistical significance with p<0.05.

In the livers from all study groups, single small foci consistent with extramedullary hematopoiesis were observed (data not shown). However, there were no quantitative differences between the groups, ranging from 1 for PBS, IL-11 and H11, to 4 for the control group.

There was no histopathologicaly evident difference in the microscopic evaluation of the lungs in all examined groups (data not shown).

### Biochemical analysis of the serum after H11 administration

The potential damage to internal organs resulting from irradiation and/or cytokine treatment was examined on day 10 by biochemical serum analyses. The results indicated no difference between irradiated and treated groups in terms of alanine transaminase (ALT), aspartate transaminase (AST) and lactate dehydrogenase (LDH) levels (Table 7).

**Table 7:** Levels of alanine transaminase (ALT), aspartate transaminase (AST) and lactate dehydrogenase (LDH) in the serum of naïve (control) mice and mice after a single irradiation and treatment with PBS, IL-11 and H11.

Treatment	Serum		
	ALAT U/L (SD)	ASPAT U/L (SD)	LDH U/L (SD)
PBS	32.0 (2.5)	54.8 (5.2)	249.0 (71.1)
IL-11	27.8 (2.6)	45.6 (7.1)	179.8 (19.7)
H11	30.2 (5.0)	48.8 (5.7)	227.6 (65.2)
Control	29.3 (8.1)	54.0 (6.0)	299.3 (16.3)

The means and SD from 5 mice per group are shown.

## Discussion

IL-11 belongs to the family of hemopoietic cytokines referred to as the IL-6-type or gp130 cytokines [20]. Its multiple biological activities are exerted by binding to cell receptors. IL-11 first binds to a specific receptor alpha (IL-11Rα) and then engages a signal transducer subunit gp130 [21]. The high-affinity IL-11 receptor complex is a hexamer consisting of two IL-11, two IL-11Rα and a homodimer of two gp130 receptors [22]. Because the gp130 protein is present on all human cell types, IL-11 activity is restricted to the cells expressing IL-11Rα. Our early studies demonstrated that the complex of IL-11/soluble IL-11Rα is functional and interacts with cells lacking membrane IL-11R [23]. However, the construction of H11 made direct targeting of gp130 possible; thus, it may induce or impede cells that normally do not sense IL-11. IL-11 was shown to be involved in hematopoiesis in different cellular and animal models [24]. Our *in vitro* results indicated that H11 activity toward CD34+Lin- cells was higher but of the same type as IL-11 stimulation [17]. However, the *in vivo* comparison of IL-11 and H11 could have different results because many types of cells could be affected by H11 following intravenous administration. Thus, a detailed *in vivo* study was needed.

Here, we compared the ability of IL-11 and H11 to reconstitute the hematopoietic system after severe damage resulting from radio/chemotherapy or radiotherapy. Their cellular, tissue and systemic effects were examined in the myelosuppressed mice. After chemo/radiotherapy, both cytokines accelerated the WBC, hematocrit and platelet recovery. The most pronounced effect was observed for platelet and WBC recoveries; however, because of the mortality of the vehicle-treated mice (PBS group), statistical evaluation was impossible. We did not investigate the reason of the deaths. However, the severe myelosuppression may result in serious adverse events such as hemorrhages and infections. Additional antibiotic treatment was effective in the deletion of the death of supralethal irradiated mice [25]. Thus, we investigated the activity of both cytokines in the less severe model of myelosuppression. After a single radiation, administration of IL-11 and H11 accelerated the recovery of WBC, hematocrit, and platelet; however, only the platelet recovery was significant compared with the PBS group. The observed systemic effect following cytokines application corresponded with a higher number of live cells in bone marrow and spleen and with the higher number of myeloid, erythroid and megakaryocyte progenitors. Our results are in agreement with previous investigations of IL-11 activity in hematopoietic reconstruction. IL-11 improved platelet nadirs and accelerated platelet recovery compared with controls in moderately to severely myelosuppressed mice and in lethally irradiated bone marrow transplanted mice [26–30]. Moreover, the significant improvements in neutrophil [29, 30] and erythroid recoveries [27] were observed after IL-11 stimulation in various models. Administration of IL-11 after supralethal irradiation promoted survival in mice, but the effect of IL-11 on the hematopoietic system was only moderate [25]. However, its hematopoietic effect was improved when other hematopoietic growth factors (TPO) or bone marrow transplantation were co-applied [25]. Similarly, when lethally irradiated mice were transplanted with syngeneic modified bone marrow cells expressing IL-11, the acceleration of recovery of circulating leucocytes, erythrocytes and platelets was indicated [31]. A study in a large animal model, in myelosuppressed cynomolgus monkeys, showed IL-11 accelerated platelet recovery compared with the controls [32]. In irradiated rhesus monkeys, IL-11 administration resulted in the acceleration of platelet and leukocyte recovery [33]. Similar to our research, the above studies indicated that the systemic effect of IL-11 administration was accompanied with the stimulation of all lineages of hematopoietic progenitors [25, 27, 29, 30, 33].

The effect of H11 was more pronounced than that of IL-11 in the radio/chemotherapy model. H11 resulted in similar, slightly higher and significantly higher numbers of WBC, HCT and PLT, respectively, than IL-11. The systemic effect was accompanied with cellularity and progenitor numbers of bone marrow and spleen in treated myelosuppressed mice. Compared with IL-11, H11 substantially increased the total number of bone marrow progenitors, especially the megakaryocyte progenitors, and also slightly increased the cellularity of bone marrow. The spleen is a hematopoietic organ in adult mice [34]; thus, we examined the activity of both cytokines in this organ. In the spleen, the activities of both cytokines differed. The kinetics of IL-11 activity was different than H11. The highest number of CFU-Mk was observed on days 15 and 30 for IL-11 and H11, respectively. Although the total number of megakaryocyte progenitors on day 15 was higher for IL-11 stimulation than for H11 at the same time point, the total number of live cells isolated from the spleen was approximately 3.4 times lower for the IL-11-treated mice compared with the H11-treated mice. Thus, these results, together with bone marrow cellularity and numbers of progenitors, ultimately resulted in better recovery (especially platelets) in the H11-treated mice.

Single irradiation resulted in less severe myelosuppression than with the radio/chemotherapy regimen, as expected. There were no systemic differences in morphology between the IL-11 and H11 treatments. However, the activity of H11 was more pronounced in the total number of bone marrow progenitors on day 10, whereas IL-11 increased the number of progenitors in the spleen on day 15. Moreover, on day 10, more late megakaryocytes were observed in the sternum of H11-treated mice than in IL-11-treated mice. This may indicate that the kinetic and action localization of both cytokines was slightly different; the effect of H11 was faster and more pronounced in the bone marrow, whereas IL-11 was more active in the spleen.

Moreover, there was a difference in applied dose between IL-11 and H11. Because the same concentration of IL-11 and H11 was used and the molecular weight of IL-11 and H11 is different (20 vs. 58.8 kDa), three times as many IL-11 molecules were available to trigger the biological effect. Accordingly, H11 activity is superior to IL-11 in reconstruction of the hematopoietic system.

As mentioned above, IL-11 belongs to the family of hemopoietic cytokines referred to as the IL-6-type or gp130 cytokines. IL-11 and IL-6 both signal through a homodimer of the ubiquitously expressed β-receptor glycoprotein 130 (gp130). First, IL-11 and IL-6 bind to an individual IL-6/IL-11 α-receptor what leads to the final complex formation with the β-receptors. Despite the similarity in receptor complex formation, both cytokines have different roles [35]. As we discussed previously at Dams-Kozlowska et al. [16] there is number of evidence that indicated that IL-6 and IL-11 bind to the different sites on gp130 what may elicit different effects (different global phosphorylation level, activation of different STAT molecules what may influence the final biological activity). The recent data by Putoczki et al. suggested that IL-11 engages pg130 differently to IL-6 [36].

Apart from Hyper-IL-11 (H11), the similar designer cytokines were constructed. Hyper IL-6 was composed of fragments of IL-6 α-receptor and IL-6 [37]. Since, both designer cytokines, H11 and Hyper-IL-6 compromise of cytokine and its cognate α receptor, they need the same receptor subunits for signal transduction. Thus, potentially they may be functionally equivalent. However, our previous data indicated that the activity of Hyper-IL-6 and H11 differed in terms of differentiation of cord blood-derived lineage-depleted CD34+ (CB Lin- CD34+) hematopoietic progenitor cells [16]. Hyper-IL-6 was more active at granulopoesis, while H11 promoted differentiation of Lin-CD34+ towards erythroid cells [16]. We did not find any data of *in vivo* activity of Hyper IL-6 in terms of hematopoiesis; all available data represented the *in vitro* or *ex vivo* studies [37–39]. Hyper IL-6 was tested *in vivo* in the process of liver cell regeneration and the acute phase response [40–42]. Thus, it is impossible to compare the activity of both fusion proteins (H11 and Hyper IL-6) in terms of *in vivo* hematopoiesis. Although, it needs to be evaluated, it was beyond the scope of this study.

Systemic administration of H11 may affect various cells of the body. Thus, we histopathologically examined internal organs such as the spleen, sternum, liver and lungs and determined selected biochemical parameters of blood. The results did not indicate a difference in the type of activity between IL-11 and H11, although quantitative and kinetic differences were observed; the most pronounced differences were observed in the spleen. An additional study is needed to explain the cause of the observed variations. In our studies, we did not observe serious adverse events following H11 treatment. It is possible, that H11 will not induce the same side effects as IL-11. However, the additional studies are needed or a strategy to properly target the designer cytokine needs to be developed. Thus, H11 may also be potentially beneficial for other IL-11-based therapies such as drug-induced hepatotoxicity [43], autoimmune encephalomyelitis (EAE) (the mouse model of multiple sclerosis MS) [44], infertility dependent on the deficiency of IL-11Rα [45] and in amelioration of cardiac fibrosis after myocardial infarction [46].

## Supporting Information

S1 Fig The measurement of the surface area of fibrosis of spleen. (PDF)
