# Supplementary material for: Effects of Designer Hyper-Interleukin 11 (H11) on Hematopoiesis in Myelosuppressed Mice
Source: PLoS One. 2016 May 4;11(5):e0154520. doi: 10.1371/journal.pone.0154520 (PMC4856347; doi:10.1371/journal.pone.0154520)
Supplement: S1 Fig — (PDF) [file pone.0154520.s001.pdf]

Supporting Information S1

The extent of fibrosis was quantified by measuring the surface area of the fibrous tissue in four different locations for each mice. The samples from three mice for each group were examined. The surface area was quantified using program CellSens (Olympus Coropration, Center Valley,PA). The obtained values (in square millimetres) were used to calculate the means and standard deviations of surface area of one single spot for all groups and means and standard deviations of total surface area of all measured fibrosis spots for each group.

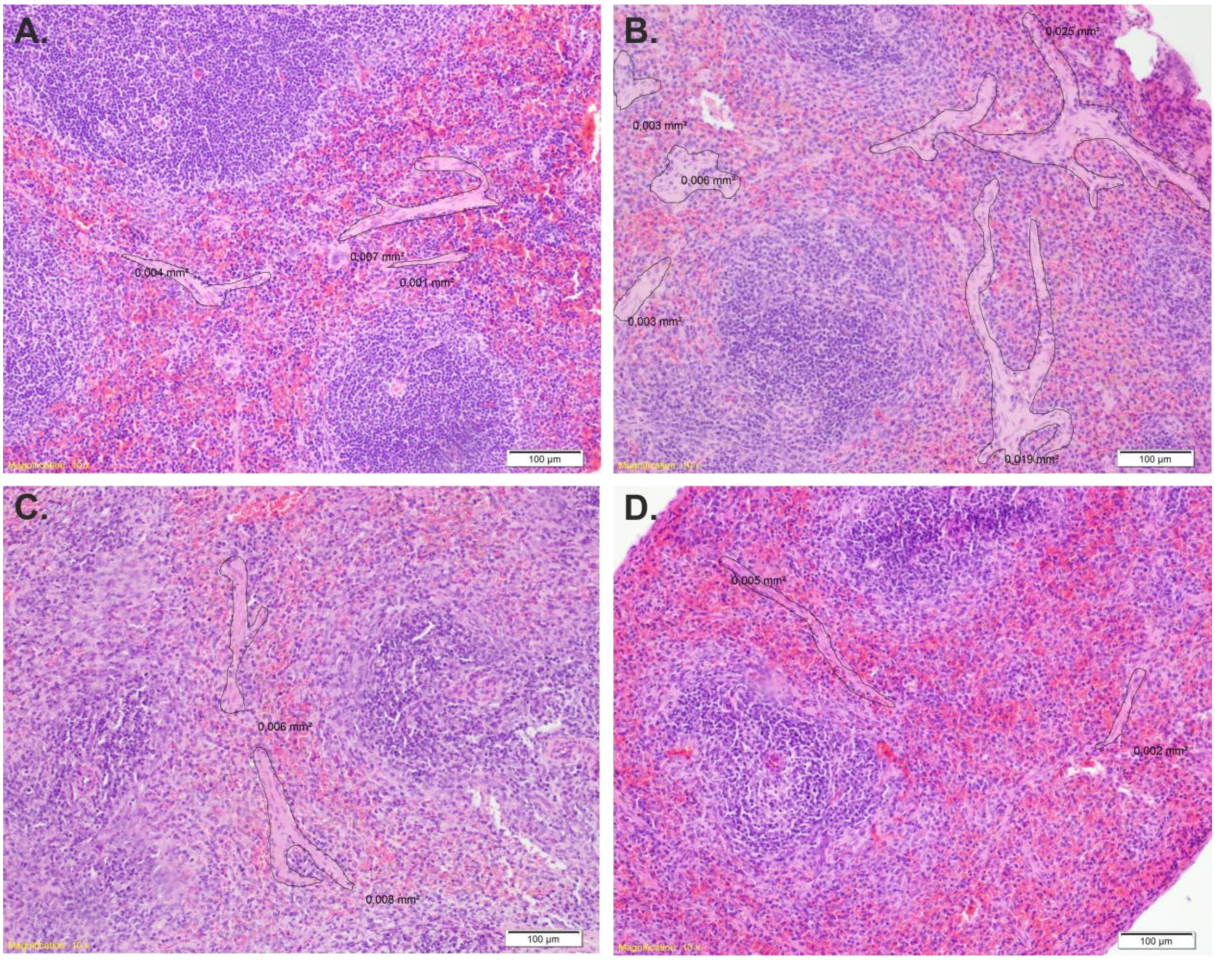

S1 Fig. The measurement of the surface area of fibrosis of spleen. A) the age-matched naïve mice (control) and the mice irradiated and then treated with B) PBS, C) IL-11 and D) H11. Ten days after treatment, spleen specimens were HE stained and examined under light microscopy (magnification 10X). Scale bar represents 100 µm.
